# Adding an App-Based Intervention to the Cognitive Behavioral Analysis System of Psychotherapy in Routine Outpatient Psychotherapy Treatment: Proof-of-Concept Study

**DOI:** 10.2196/35482

**Published:** 2022-08-09

**Authors:** Anna-Lena Netter, Ina Beintner, Eva-Lotta Brakemeier

**Affiliations:** 1 Department of Clinical Psychology and Psychotherapy Philipps University of Marburg Marburg Germany; 2 MindDoc Health GmbH Munich Germany; 3 Department of Clinical Psychology and Psychotherapy University of Greifswald Greifswald Germany

**Keywords:** Cognitive Behavioral Analysis System of Psychotherapy, persistent depressive disorder, blended therapy, internet and mobile-based Interventions, routine clinical care, eHealth, mobile phone

## Abstract

**Background:**

The Cognitive Behavioral Analysis System of Psychotherapy (CBASP) is an empirically supported psychotherapeutic treatment developed specifically for persistent depressive disorder. However, given the high rates of nonresponse and relapse, there is a need for optimization. Studies suggest that outcomes can be improved by increasing the treatment dose via, for example, the continuous web-based application of therapy strategies between sessions. The strong emphasis in CBASP on the therapeutic relationship, combined with limited therapeutic availabilities, encourages the addition of web-based interventions to face-to-face therapy in terms of blended therapy.

**Objective:**

The aim of this study was to test an app-based intervention called *CBASPath*, which was designed to be used as a blended therapy tool. *CBASPath* offers 8 sequential modules with app-based exercises to facilitate additional engagement with the therapy content and a separate exercise to conduct situational analyses within the app at any time.

**Methods:**

*CBASPath* was tested in an open pilot study as part of routine outpatient CBASP treatment. Participating patients were asked to report their use patterns and blended use (integrated use of the app as part of therapy sessions) at 3 assessment points over the 6-month test period and rate the usability and quality of and their satisfaction with *CBASPath*.

**Results:**

The results of the pilot trial showed that 93% (12/13) of participants used *CBASPath* as a blended tool during their therapy and maintained this throughout the study period. Overall, they reported good usability and quality ratings along with high user satisfaction. All participants showed favorable engagement with *CBASPath*; however, the frequency of use differed widely among the participants and assessment points. Situational analysis was used by all participants, and the number of completed modules ranged from 1 to 7. All participants reported blended use, although the frequency of integration in the face-to-face sessions varied widely.

**Conclusions:**

Our findings suggest that the digital augmentation of complex and highly interactive CBASP therapy in the form of blended therapy with *CBASPath* is feasible in routine outpatient care. Therapeutic guidance might contribute to high adherence and increase patient self-management. A few adjustments, such as saving entries directly in the app, could facilitate higher user engagement. A randomized controlled trial is now needed to investigate the efficacy and added value of this blended approach. In the long term, *CBASPath* could help optimize persistent depressive disorder treatment and reduce relapse by intensifying therapy and providing long-term patient support through the app.

## Introduction

### Background

Up to 30% of all depressive disorders take a chronic course [1,2]. Persistent depressive disorder (PDD) is, compared with nonchronic major depressive disorder, associated with an earlier onset, a longer duration of the disorder, higher comorbidity rates of axis 1 and axis 2 psychiatric disorders, higher rates of suicidal behavior, alexithymia [3], and more childhood maltreatment [4] Not surprisingly, treatment outcomes—both pharmacological and psychotherapeutic—are poorer, and recurrences are higher [5], often resulting in a higher frequency of treatment seeking [3]. Specific challenges in the treatment of PDD include impaired interpersonal functioning (ie, a more submissive and hostile interpersonal style), behavioral and emotional avoidance, pronounced help and hopelessness, rigid behavioral patterns, and high personal distress [3,6,7].

The *Cognitive Behavioral Analysis System of Psychotherapy* (CBASP), originally developed by McCullough [8], is, to date, the only psychotherapeutic approach that specifically targets PDD. CBASP integrates cognitive behavioral therapy (CBT) with interpersonal and psychodynamic theories and strategies [9]. The central element of treatment [10] is CBASP-specific *situational analysis*, a highly structured, multi-step interpersonal problem-solving task through which patients learn that their behavior has consequences and how to relate functionally to others. Situational analysis includes behavioral training in the form of role-playing, and the Kiesler interpersonal circumplex model [11] is used as a supplemental interpersonal strategy. At the start of therapy, formative early learning experiences are collected by creating a list of *significant others’ histories*. The significant others history is then related to current interpersonal problems, initially within the therapeutic relationship. To achieve this, therapists strive for a therapeutic alliance described by *disciplined personal involvement*. Therapists regularly reveal their own feelings and reactions to patients’ behavior and thus provide the foundation for corrective, healing interpersonal experiences within the therapy setting. Differences between the therapists’ responses and negative experiences with significant others of the patients are emphasized using the *interpersonal discrimination exercise* (IDE).

CBASP as an outpatient treatment is an empirically supported treatment [12-15]. It has been shown to be particularly beneficial for patients with early onset [12], childhood maltreatment [16,17], and in combination with medication [14]. CBASP has also been shown to be effective in inpatient settings in open pilot studies [18-20]. However, high rates of nonresponse (40%-60%), nonremission (60%-80%), and relapse (up to 50% after 2 years) indicate the need for optimization [13,15,18,21].

Studies have shown that (1) a larger number of therapy sessions (at least 18 sessions [22]); (2) a longer treatment duration [15,22,23]; and (3) an intensification of CBASP in the sense of a dose increase through, for example, additional group therapy or another additional therapy program [18,20] might improve therapy outcomes.

Psychotherapy resources are limited, and as CBASP requires special training, few therapists offer this treatment in routine care. Increasing CBASP therapy sessions for patients during treatment would thus result in much longer waiting times for individuals seeking this treatment. Hence, this is neither practicable nor efficient, and solutions are needed to increase the treatment dose without increasing the number of treatment sessions. This solution could then also be useful in supporting patients to maintain their treatment gains in the long term.

Internet- and mobile-based interventions (IMIs) offer high potential for psychotherapy [24]. Desktop-based IMIs have been proven to be effective and cost-efficient in delivering mental health care in numerous trials [25-27]. Guided interventions are associated with better adherence and outcomes than unguided ones [27-30]. Smartphone- or app-based IMIs have been less researched but promise to yield small to moderate effects in the treatment of depression (*g*=0.33 and *g*=0.56 [31,32]).

Research findings suggest that IMIs may also improve face-to-face therapy. In *blended therapy*, face-to-face therapy is augmented with IMIs [33]. This option of increasing the effectiveness of conventional therapy has been shown to be feasible [33-36]. The superiority of blended therapy compared with standard psychotherapy has been shown in 2 studies for mild to moderate depression, also at the 6-month follow-up [36,37]. A web-based self-management program, in combination with care as usual, also showed promising results for recurrent depression [38]. A plausible explanation for this large effect may simply be that the treatment dose was increased by adding the IMI. Another explanation could be that the more specific effect of self-directed, between-session practice and application of therapy skills in daily life contributed a significant additional benefit to an already effective therapy [39].

When conceptualizing IMIs within CBASP, the highly structured nature of the treatment and the high relevance of the therapeutic relationship but the simultaneous limitation of therapeutic availabilities suggest a blended approach to meet the needs of PDD patients. Interpersonal strategies for shaping the therapeutic relationship and behavioral training can still be applied in face-to-face sessions, whereas an IMI could support patients to elaborate and continuously apply learned CBASP strategies (eg, situational analysis) in everyday life, thus increasing the therapy dose. A total of 2 case reports of internet-based situational analysis training after CBASP inpatient treatment indicated good acceptability and feasibility [40]. Both individuals found the training helpful in transferring therapy content to everyday life.

### Objective

Building on these positive first experiences, we developed an app-based intervention called *CBASPath* to be used as part of a blended CBASP therapy. The aim of this paper is to introduce the features of *CBASPath*, describe the blended approach, and present the results of a pilot study investigating the feasibility of *CBASPath* use in routine clinical care. We examine the participants’ engagement with *CBASPath*, which was blended with face-to-face sessions. Usability, app quality, and user satisfaction are important factors influencing continuous use [41]. Therefore, an additional open research question targeted participants’ perceived usability, quality ratings, and satisfaction. Depression severity was observed in an exploratory manner.

## Methods

### Overview

To examine the feasibility of the blended *CBASPath* intervention, a single-arm, open pilot study was conducted in a routine care setting. Data were collected over a 6-month period at 4 assessment points.

### Ethics Approval

The ethics committee of the Philipps University of Marburg granted ethical approval for all study procedures (file number 2019-29k).

### Procedures

CBASP-certified practitioners in Germany were invited by mail to integrate *CBASPath* in their ongoing CBASP treatments in the context of the pilot study. Interested therapists received information about *CBASPath* and were instructed on how to give their patients access to it. Participants were given a link to a web-based survey [42]. Before the start of the first survey, participants were informed about study participation and app use and signed an informed consent form. Invitations for subsequent assessments were sent via email. Data were collected pseudonymously using self-generated codes to allocate assessment points. Demographic variables, self-reported diagnoses, depression severity, and prior experience with psychotherapy, as well as information on participants’ current therapy and participants’ attitudes toward IMIs, were assessed at baseline. Participants’ engagement in *CBASPath* and depression severity were assessed 6, 12, and 24 weeks after initial use. Usability, app quality, and satisfaction with the app were assessed at week 12. A raffle of web-based vouchers for participants who took part in all the surveys served as an incentive. Manuals for therapists and patients provided suggestions for incorporating the app into the therapy. Telephone consultations for therapists were offered as needed, primarily related to study procedures and technical difficulties.

### Participants

German-speaking patients who were aged at least 18 years and who were currently undergoing outpatient CBASP therapy (regardless of the stage of therapy) were eligible to participate. Additional inclusion criteria were the possession of a smartphone with internet access (Android or iOS operating system) and sufficient skills to use it, a valid email address, and willingness to take part in the web-based survey.

### The CBASPath Intervention and Its Use in Blended CBASP Therapy

*CBASPath* is a CBASP-specific mobile app course integrated into the *MindDoc* app (MindDoc Health GmbH). *MindDoc* is a certified class 1 medical device that includes an adaptive monitoring system, automated feedback about the user’s mental health, and courses and exercises facilitating the self-management of mental health complaints.

For the study, a designated setup of the *MindDoc* app was created, including the *CBASPath* material and additional content depending on the individual symptoms (psychoeducation about depression, mindfulness, relaxation, rumination, self-compassion, and sleep). All content could be used at the user’s discretion.

The *CBASPath* material was developed specifically for this study and was accessible only to the study participants. All contents were based on the McCullough [8] concept for outpatient CBASP therapy, related treatment manuals [43,44], and a self-help book for patients with PDD [45]. We also included both CBASP practitioners and patients in the development process.

**Figure 1:**
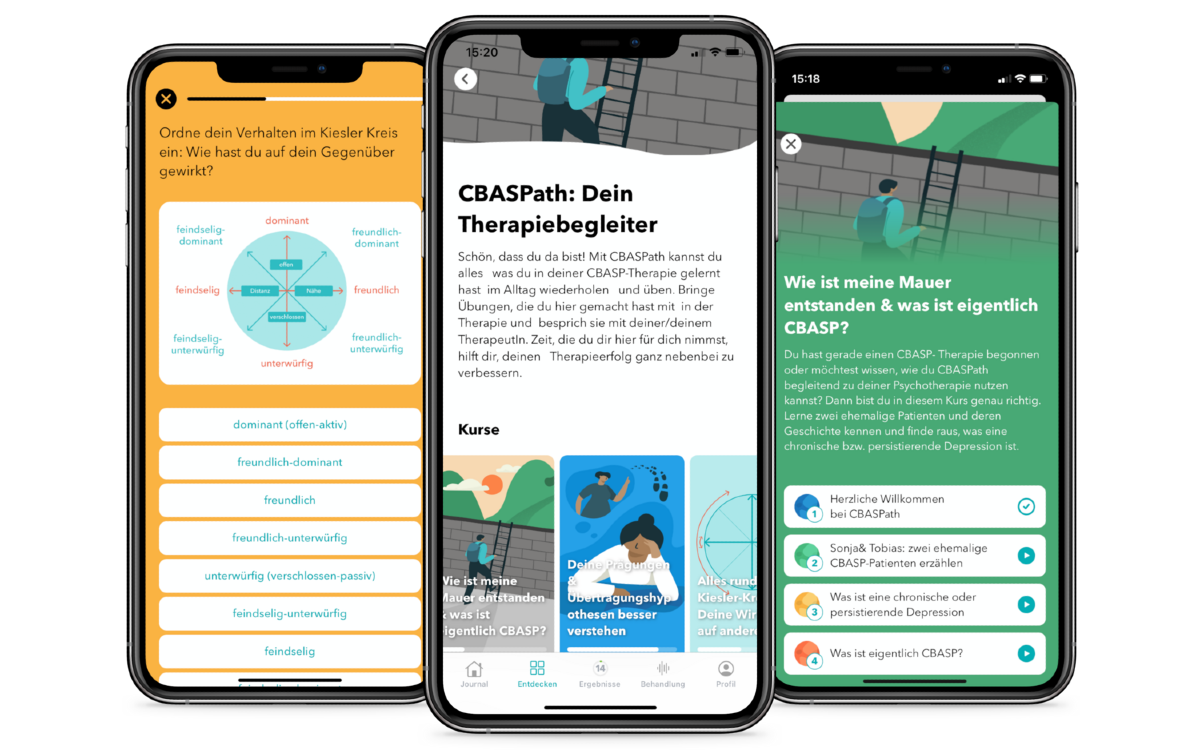
User interfaces of the *CBASPath* course from left to right: (1) Categorizing one’s own behavior in the Kiesler circumplex model in the context of a personal situational analysis; (2) home screen of the *CBASPath* course; and (3) overview of different exercises in module 1.

We strived to make all content as app-friendly as possible; for example, by keeping reading times short, avoiding unnecessary typing, and using interactive features. *CBASPath* includes 8 sequential modules in line with CBASP therapy and an additional module comprising 4 different step-by-step exercises for conducting personal situational analysis (interpersonal, future, and internal focus) and IDE at any time (for a detailed description, see Table 1 and sample screenshots in Figure 1). Minor adjustments to the original situational analysis and IDE exercises were made to simplify their use on the smartphone (eg, describing the situation using a meaningful heading and using multiple-choice answer options where possible).

The *CBASPath* intervention serves as an augmentation to face-to-face CBASP therapy. Although the course is primarily designed for patients to use on their own between sessions, individual exercises are closely intertwined with the content of CBASP therapy. Patients are repeatedly encouraged to discuss difficulties, success, and results of exercises with their therapist in sessions, or therapy content are followed up with the help of specific exercises (eg, on transference hypothesis). The extent to which *CBASPath* is embedded into therapy sessions can be adapted according to individual needs and the therapy stage. Intensive therapeutic guidance at the beginning in the form of a detailed introduction, specific time for questions or doubts, and concrete suggestions for suitable exercises to work on between sessions might motivate patients and prevent early attrition. Although all 8 modules are designed to be completed between sessions without therapeutic help, not reviewing completed exercises, as with analog homework, could be demotivating and have a negative impact on future use [46]. Blended use might provide additional opportunities for disciplined personal involvement, IDE, and eventually corrective relationship experiences (eg, recognition through the promotion of completed exercises and dealing with problems and doubt). Over the course of therapy, *CBASPath* should become an integral part of therapy, and therapeutic guidance can be gradually faded out. After the therapy is completed, *CBASPath* can serve as a self-help tool to maintain therapeutic gains and prevent relapse. Ideally, the course is now a daily companion for the patient, which they can fall back on as needed and thus continue to incorporate CBASP skills into everyday life.

**Table 1:** Comparison of Cognitive Behavioral Analysis System of Psychotherapy (CBASP) components and their representation within *CBASPath*.

CBASP therapy components (disciplined personal involvement)	CBASPath content
Therapy start	Module 1: Information on the blended use of *CBASPath*; psychoeducation on persistent depressive disorder and CBASP; written and audio-based introduction of 2 prototype patients
Significant others history and transference hypotheses	Module 2: Psychoeducation on significant others history and transference hypotheses including examples of prototype patients; reflecting and journaling of personal significant others history and transference hypotheses developed in therapy
Kiesler circumplex model	Module 3: Psychoeducation; solidifying knowledge with interactive exercises (eg, experiencing different dimensions of the model through short videos or becoming familiar with the model by positioning celebrities in the model); hands-on exercise with reflection (eg, to try out different and unfamiliar behaviors in everyday life and to record the reaction of others in the app)
Situational analysis and training of interpersonal skills	Module 4: Psychoeducation on situational analysis; step-by-step training based on a prototype patient’s situation presented via video and subsequent sample solutions. Module 6: Video-based empathy training; hands-on empathy exercise with reflection Situational analysis exercises: conducting personal situation analysis (3 different types of situation analysis with interpersonal, future, and internal focus)
IDE^a^	Module 5: Psychoeducation on IDE and “hot spot” situations; IDE training based on a prototype patient’s situation and subsequent sample solutions; reflecting and journaling of personal hot spots. IDE exercise: conducting personal IDEs
Therapy completion	Module 7 and 8: Summarizing (and celebrating!) personal therapy successes; reflecting on helpful therapy skills as part of relapse prevention; planning further use of app as long-term support and maintenance

^a^IDE: interpersonal discrimination exercise.

### Measures

Participants’ engagement in the *CBASPath* was measured using self-report items at weeks 6, 12, and 24. Participants specified their average duration per app use in minutes, frequency of use per week, whether they conducted situational analysis and IDE, and how often *CBASPath* was used during face-to-face sessions as an additional therapy tool or for prediscussion and follow-up discussion of completed content, all regarding the last measurement time. The usability of the *CBASPath* course was measured using the System Usability Scale (SUS) [47]. The scale, which has high validity and economy, was adapted to the app context for the purpose of this study, as recommended by the author [47]. Participants’ global satisfaction with the blended use of *CBASPath* was assessed using the Client Satisfaction Questionnaire adapted for internet-based Interventions (CSQ-I) [41]. Good construct validity and high internal consistency have been demonstrated [41]. The Mobile App Rating Scale [48] is the most frequently used scale for evaluating the quality and content of mental health apps. The German translation of the user version of Mobile App Rating Scale (uMARS) [49] used in this study includes 4 objective subscales (engagement, functionality, aesthetics, and information quality) and one subjective quality scale. The uMARS has good internal consistency and test–retest reliability [49]. In addition, the 16-item Attitudes toward Psychological Online Interventions Questionnaire (APOI) [50], was used to assess the participants’ general attitudes toward IMIs, including a total score and 4 subscales (skepticism and perception of risk, confidence in effectiveness, technologization threat, and anonymity benefits), with higher scores indicating a more positive attitude. Depression severity was assessed using the Beck Depression Inventory–Second Edition [51].

### Statistical Analysis

Owing to the small sample size, all data collected are presented and compared at the individual case level. Mean values and SDs were calculated for the expectations toward web-based interventions, usability and quality ratings, and user satisfaction. Analyses were performed using IBM SPSS Statistics (version 27.0; IBM Corporation) for Windows. Owing to the small sample size and the fact that data on use intensity varied greatly, participants’ engagement in *CBASPath* use was categorized into high, medium, and low use. High use was rated as at least twice weekly app use with a duration of at least 15 minutes of situational analysis use and at least five completed modules. Medium use was rated as once or twice a week with at least 5 minutes of situational analysis use and at least one completed module. Below this level of use was classified as low. Individual values on depression severity were visualized in a scatter plot.

## Results

### Participants

A total of 18 participants registered for the pilot study at baseline, of whom 5 (28%) did not participate in any further measurement time point and were therefore handled as dropouts. Another participant was excluded from data analysis as he could not download the study version of the app and could therefore not use *CBASPath*. The final study sample included 12 participants, and the final survey in week 24 was completed by 11 (92%) participants. Table 2 summarizes the baseline sociodemographic and clinical characteristics of all participants. The sample was heterogeneous in terms of age, gender, and level of education. All patients reported depressive disorder as the treatment diagnosis. Of the 12 participants, 9 (75%) reported at least one completed psychotherapeutic treatment. All participants were in different stages of outpatient CBASP therapy when they began using the app; 50% (6/12) received CBASP group therapy, and 67% (8/12) had been in ongoing therapy for more than a year. Of the 12 participants, 10 (83%) perceived their current treatment as helpful, and 2 (17%) were unsure. At the end of the study period after 24 weeks, of the remaining 11 participants, 3 (27%) reported having completed their outpatient treatment, and the rest were still in treatment. None of the participants reported a self-help experience with IMIs; 33% (4/12) reported a self-help experience with books. Participants’ general attitudes toward IMIs can be considered positive with an average APOI total score of 48.33 (SD 3.82, range 41.00-56.00). Participants’ individual APOI total scores and subscale scores are listed in Multimedia Appendix 1.

**Table 2:** Participants’ sociodemographic and clinical characteristics at baseline^a^.

Participant	Age (years)	Sociodemographic data	Treatment diagnosis	Duration of the current episode (years)	Age of onset (years)	Comorbid disorders	Previous PT^b^	Setting and duration of current CBASP^c^ PT	Current PT helpful
Participant 1	54	Male, married; 3 children; university degree; full-time job	MDD^d^	6	39	Dysthymia and personality disorder	More than 3 outpatient PT and 2 inpatient PTs	Outpatient group PT for >12 months	Unsure
Participant 2	46	Female; firm partnership; lower secondary education; on sick leave	MDD	4	33	None	1 outpatient PT and 1 inpatient PT	Outpatient PT for 6 to 12 months	Unsure
Participant 3	32	Female, divorced; lower secondary education; full-time job	RDD^e^	4	29	None	1 inpatient PT	Outpatient group PT for >12 months	Yes
Participant 4	27	Male; single; university degree; full-time job	MDD	8	23	None	1 outpatient PT and 1 inpatient PT	Outpatient PT for less than a month	Yes
Participant 5	32	Male; married; 1 child; upper secondary education; full-time job	Other (emotional instability)	5	21	None	None	Outpatient PT for >12 months	Yes
Participant 6	30	Male; single; upper secondary education; full-time job	MDD	6	17	None	2 outpatient PTs and 1 inpatient PT	Outpatient group PT for >12 months	Yes
Participant 7	30	Female; firm partnership; university degree; full-time job	Dysthymia	6	17	Social phobia	None	Outpatient group PT for >12 months	Yes
Participant 8	40	Female; married; lower secondary education; retired	RDD	6	17	Personality disorder and chronic pain	2 outpatient PTs and 1 inpatient PT	Outpatient PT for >12 months	Yes
Participant 9	33	Female; single; lower secondary education; not employed	Dysthymia	2	15	RDD and social phobia	3 outpatient PTs	Outpatient day clinic for less than a month	Yes
Participant 10	22	Female, single, upper secondary education, full-time job	Dysthymia	6	13	None	None	Outpatient group PT for >12 months	Yes
Participant 11	46	Male; firm partnership; university degree; full-time job	Dysthymia	4	—^f^	None	1 outpatient PT	Outpatient PT for 6 to 12 months	Yes
Participant 12	62	Male; married; 3 children; lower secondary education; retired	RDD	5	15	None	None	Outpatient group PT for >12 months	Yes

^a^All data presented are self-reported; education level according to the International Standard Classification of Education.

^b^PT: psychotherapy treatment.

^c^CBASP: Cognitive Behavioral Analysis System of Psychotherapy.

^d^MDD: major depressive disorder.

^e^RDD: recurrent depressive disorder.

^f^Not available.

### General Usability, Quality Ratings, and User Satisfaction of the CBASPath Course

*CBASPath*’s overall usability was rated with a mean total SUS score of 85.21 (SD 10.74, range 57.50-97.50) on a 100-point scale. Of the 12 participants, 8 (67%) rated *CBASPath*’s usability as excellent (SUS ≥85.5), 3 (25%) as good (SUS ≥71.4), and 1 (8%) participant rated the usability as *ok* (SUS ≥50.9) [52]. Participants’ average satisfaction with the *CBASPath* resulted in a mean CSQ-I score of 28.00 (SD 2.26, range 24-31) out of 32 scale points, indicating high user satisfaction [41]. Users’ quality ratings of *CBASPath* resulted in an average uMARS total score of 3.99 (SD 0.30, range 3.51-4.56; 1=poor to 5=excellent), indicating good quality. Approximately equally high scores were found for the subscales *function* (mean 4.10, SD 0.55, range 3.00-5.00), *aesthetics* (mean 4.03, SD 0.30, range 3.33-4.33), and *information quality* (mean 4.04, SD 0.32, range 3.50-4.50). The engagement subscale was rated at 3.80 (SD 0.50, range 3.00-4.60), and subjective app quality was 3.67 (SD 0.29, range 3.00-4.00). The individual SUS, CSQ-I, and uMARS ratings of all participants are presented in detail in Multimedia Appendix 2.

### Participants’ Engagement With the App and in the Blended App Use Setting

All 12 participants reported using the *CBASPath* throughout the study period. Of the 12 participants, 9 (75%) reported using situational analysis within the first 6 weeks of use; as the study progressed, all participants reported using situational analysis. In an open response field at the end of the survey, 25% (3/12) of participants noted that they had found the situational analysis particularly helpful (participants 5, 6, and 7). None of the participants reported IDE use at 6 weeks; 8% (1/12) stated using it at week 12 (participant 1), and 42% (5/12) reported IDE use at 24 weeks (participants 1, 4, 5, 6, and 8). Participants reported using *CBASPath* at least once a week to daily for 1 to 50 minutes at a time, with wide variation between individual participants and within measurement time points (see Table 3 for variation of frequency of use during the study period). The most intensive use was reported at week 12: 75% (9/12) reported using *CBASPath* at least three times a week. They completed between 1 and 7 out of the 8 available CBASP modules. Owing to the small sample size and the fact that participants’ engagement in CBASPath and blended use varied greatly, the individual use patterns were categorized into low to high use; of the 12 participants, high use could be found in 7 (58%) participants (participants 1, 2, 4, 5, 8, 9, and 12), medium use was shown by 5 (42%) participants (participants 3, 6, 7, 10, and 11), and none showed low use. All participants reported some form of blended use during the 24-week study period; however, the frequency of integration into therapy varied widely between individual participants. For example, 17% (2/12) of participants (participants 1 and 10) reported blended use in nearly every session, whereas others reported consistent blended use (participants 4 and 6) or less frequent involvement, totaling approximately 2 to 4 times (participants 12 and 11). Individual engagement in *CBASPath* and the blended use of all participants can be found in detail in Table 3.

**Table 3:** Participants’ *CBASPath* use categorized as medium and high adherence and reported blended use^a^.

MTP^b^			Completed Modules, n		Duration per app use in minutes							Days of app use per week							Situational analysis use							Interpersonal discrimination exercise use							Blended use				
					Week 6^c^		Week 12^d^		Week 24^e^		Week 6			Week 12		Week 24		Week 6			Week 12		Week 24		Week 6			Week 12		Week 24		Week 6			Week 12		Week 24
**High adherence**																																					
	Participant 1	6		10		10		5		1			Daily		Daily		Yes			Yes		Yes		No			No		Yes		In every session			In >5 sessions		In every session	
	Participant 2	5		50		15		15		Daily			Daily		5		Yes			Yes		Yes		No			No		No		In 1 to 2 sessions			Not at all		In 3 to 5 sessions	
	Participant 4	5		20		20		15		2			2		3		No			Yes		Yes		No			No		Yes		In 1 to 2 sessions			In 1 to 2 sessions		In 3 to 5 sessions	
	Participant 5	5		20		20		15		4			2		—^f^		Yes			Yes		Yes		No			No		Yes		In 3 to 5 sessions			In 1 to 2 sessions		In 3 to 5 sessions	
	Participant 8	6		15		20		30		1			2		2		Yes			Yes		Yes		No			No		Yes		In 1 to 2 sessions			3 to 5 times		In 1 to 2 sessions	
	Participant 9	7		30		30		—		4			4		—		Yes			Yes		Yes		No			Yes		—		In 3 to 5 sessions			In 1 to 2 sessions		—	
	Participant 12	7		35		15		15		2			3		1		Yes			Yes		Yes		No			No		No		In 1 to 2 sessions			Not at all		In 1 to 2 sessions	
**Medium adherence**																																					
	Participant 3	3		2		5		5		1			Daily		1		Yes			Yes		Yes		No			No		No		In >5 sessions			In 3 to 5 sessions		In >5 sessions	
	Participant 6	1		1		12		15		0			4		3		No			Yes		Yes		No			No		Yes		In 1 to 2 sessions			In 1 to 2 sessions		In 3 to 4 sessions	
	Participant 7	4		30		5		10		5			Daily		1		Yes			Yes		Yes		No			No		No		In 1 to 2 sessions			In 1 to 2 sessions		In every session	
	Participant 10	2		10		10		15		2			5		2		Yes			Yes		Yes		No			No		No		In every session			In every session		In every session	
	Participant 11	2		20		1		10		1			Daily		1		No			Yes		Yes		No			No		No		Not at all			In 1 to 2 sessions		In 1 to 2 sessions	

^a^Participants’ self-reported adherence was categorized as medium and high use regarding data on completed modules, duration per app use, frequency of app use per week, and situational analysis use.

^b^MTP: measurement time point.

^c^Ratings of week 1 to 6 after initial use of *CBASPath*.

^d^Rating of week 7 to 12 after initial use of *CBASPath*.

^e^Ratings of week 13 to 24 after initial use of *CBASPath*.

^f^Missing data.

### Depression Severity

**Figure 2:**
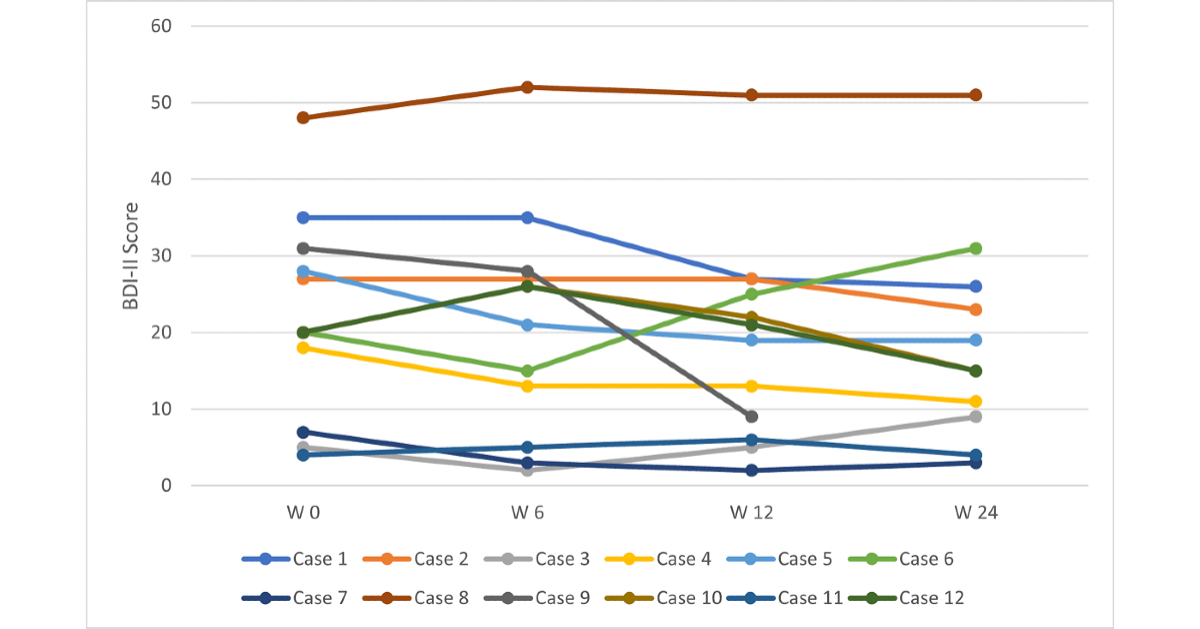
Participants’ individual BDI-II scores over the course of the study period. BDI-II: Beck Depression Inventory-Second Edition; W: week.

Figure 2 illustrates the Beck Depression Inventory–Second Edition total scores of the 12 patients at the start of *CBASPath* use and during the course of the study. The trajectories vary considerably. Of the 12 participants, 3 (25%; participants 3, 7, and 11) had very low depression scores at the beginning of the study, which also remained at a low level during the study period. Participant 8 showed high depression scores throughout the study period, and the depression severity of participant 6 increased after a slight improvement in the sixth week. The remaining participants (1, 2, 4, 5, and 12) showed a slight improvement in symptoms over the course of the study. Owing to pseudonymous participation, the dropout reason for participant 9 is unclear.

## Discussion

### Principal Findings

The purpose of this study was to outline the concept and feasibility of a blended CBASP treatment in a routine care setting and investigate the usability and quality of and satisfaction with the app-based *CBASPath* course.

Our findings suggest that the digital augmentation of rather complex and highly interactive CBASP therapy in the form of blended therapy is feasible in routine outpatient care. Participants reported continuous blended use over the course of the study, good usability and quality ratings, and high user satisfaction. The positive experiences of conducting situational analysis on the web reported in another pilot study by Brakemeier et al [40] can, therefore, be extended to the app context and blended setting in this pilot study.

### Usability of, Quality of, and Satisfaction With CBASPath

The good to excellent usability ratings indicate that working with *CBASPath* content on a smartphone is feasible and that disadvantages compared with computer-based IMIs, such as the small screen and different use patterns (eg, lower rates of user engagement), could be well compensated for by an adapted design [31]. For example, the design of the exercises was adjusted to minimize typing by using a multiple-choice format instead of text entries. To assist users who are less technology savvy in getting started, all patients were provided with written instructions for using the app, suggesting possibilities for blended use and answers to frequently asked technical questions (eg, “how do I take a screenshot?”).

The quality rating underlines the high usability of *CBASPath*. The app’s functionality (including performance, ease of use, navigation, and gestural design), aesthetics (layout, graphics, and visual appeal of the app), information provided via the app (quality, quantity, visual information, and credibility of the source), and opportunities for participant engagement (entertainment, interest, customization, and target group) were unanimously rated positive, as was subjective app quality and user satisfaction, All 12 participants would continue to use *CBASPath* by themselves and would recommend it to others; however, almost all were hardly willing to pay for the app. Considering that psychotherapy is covered by health insurance in Germany, that some health apps can be prescribed by health professionals, and that participants perceived their current therapy as mainly helpful, their willingness to spend additional money might have been limited.

*CBASPath* was found to be helpful by most participants in dealing with their difficulties, and they would use it again if they needed help, which again underlines the high satisfaction. A benefit through the increase of self-management skills by having independent access to digital therapy content, as found in a former study on blended therapy acceptance [53], might apply to CBASP as well and foster patients’ autonomy.

### Participants’ Engagement With CBASPath and Its Blended Use

Participants’ medium to high engagement with *CBASPath* and the reported blended use support the feasibility and acceptance of the presented blended modification of CBASP therapy.

The patient who did not use *CBASPath* could not install the app because of an outdated operating system on his smartphone and was therefore excluded from further analysis. *CBASPath* was continuously used over a 6-month period by at least 92% (11/12) of participants. Another participant did not participate in the final survey at the end of the 6-month study period; thus, we could not specify his use at the end of the study.

The good acceptance of blended CBASP therapy among patients is further reinforced by the fact that none of the patients in this study reported previous IMI experience but nevertheless showed sufficient user engagement, although prior experience with eHealth is associated with higher acceptance of digital applications [54]. Although the validity of the reported use data is reduced because of the small sample size and self-reported data, it supports previous findings that therapeutic guidance for IMIs can lead to high adherence and might even reduce the risk of treatment dropout [28,33,55].

It is particularly encouraging that all participants reported using the app-based situational analysis and considered it particularly helpful, as situational analysis is a central component of CBASP therapy [43,44]. Situational analyses created in the app can be reinforced by behavioral training during the session. Moreover, good situational analysis skills were associated with better treatment outcomes in a previous study [56]. The smartphone, as a daily object, seems to be a feasible device and therefore particularly promising for transferring central CBASP skills, such as situational analysis, to patients’ everyday life.

The benefit of CBASP-specific modules remains unclear as their extent of use varied widely. Most participants (8/12, 67%) had completed at least half of the 8 available modules by the end of the study period. Participants were in different stages of therapy when they started using *CBASPath*, which is why some of the modules might not have suited the respective therapy stage so that they were no longer or not yet used (eg, experienced patients may already be very familiar with their significant other’s history and transference hypothesis and therefore no longer need module 2). In addition, longer reading times of some exercises of up to 15 minutes might have been an additional barrier. Brief skill-based app content is related to high and long-lasting use [57], as it reflects the typical short but frequent use of smartphones. The first 2 *CBASPath* modules could be expanded by the inclusion of hands-on exercises that prioritize *getting into the action* in addition to journaling session content and psychoeducation at a minimum. Furthermore, the low blended use of the modules because of a very flexible approach on how to embed *CBASPath* into therapy may have led therapists to recommend the modules more for independent use than incorporating them into the session. For instance, the blended use of situational analysis was advised in the written information that patients and therapists received, and modules, by contrast, were designed as self-help to bridge sessions and might therefore be less integrated into sessions. It is plausible that, in some cases, only a few modules were completed, but situational analysis was still used regularly within the blended setting.

Therapists also did not receive training for blended therapy in addition to written information; the offered telephone support was used by only one of the therapists. Training for therapists could be another way of optimizing blended use and thus improving the uptake of modules [58]. It is also suggested that the effectiveness of IMIs depends on the long-term use of an app, which can be promoted through face-to-face sessions [59].

The participants’ feedback on *CBASPath* revealed options for further technical improvements that could lead to higher engagement with the course material. For example, an option to save entries directly in the app for future reference (instead of taking screenshots) was mentioned by several users. The use of voice input may have been additionally beneficial, especially for the frequently used situational analysis exercises.

### Limitations

Several limitations should be considered. Data were available only for patients who used the app. Therefore, reasons for dropout or nonuse of *CBASPath* could not be assessed, and the present results could be positively biased. Considering that participants’ overall attitudes toward IMIs was rather positive, there might be a selection bias, as patients with a high general acceptance of IMIs might have been interested in participating in the study and might therefore have been particularly motivated to use the app. However, none of the participants reported experiences with blended use, and participants’ general attitudes toward IMIs were similar compared with a sample with mild to moderate depression (mean 48.33 compared with mean 48.3) [50]. In addition, an affinity for smartphone use was an inclusion criterion. For more patients who are skeptical or less technologically savvy, blended use could pose additional challenges.

Furthermore, the reliability and generalizability of the results were limited because of the small sample size. Results regarding engagement and adherence should be viewed with caution because of the self-reported nature of the data collected. Objective use data should be used in subsequent studies.

Finally, there was no control of how exactly *CBASPath* was embedded in face-to-face therapy; thus, the form of blended use can vary greatly between individuals. Therefore, a subsequent randomized controlled trial (RCT) should compare manualized blended therapy with *CBASPath* with therapy without app support.

### Implications and Future Directions

Overall, good usability and quality ratings, high user satisfaction, and favorable adherence to user engagement and blended use are good prerequisites for further adaption, a subsequent RCT on the efficacy, and implementation of the blended CBASP therapy concept in different routine care settings such as CBASP outpatient therapy.

A recently published RCT comparing immediate and long-term effectiveness [36] found that blended therapy could have an additional positive effect on psychotherapy for depression in terms of symptom reduction, improved therapeutic processes, and higher health-related quality of life. As an increase in therapy dose and duration seems beneficial to further improve CBASP therapy [15,20,22,60], subsequent RCT studies should further investigate whether blended CBASP therapy is also beneficial and should therefore be implemented. Future studies should include long-term follow-up assessments to evaluate whether long-term stabilization of symptoms can be achieved.

The reported use patterns and concurrent blended use appeared to be contrary in some cases. For example, when comparing weeks 12 and 24, participants 2, 3, 5, 6, 7, and 12 reported an increase in blended use during face-to-face therapy, although their user engagement with the app decreased. Therefore, further research should investigate which frequency and intensity of blended use are most effective and efficacious for different stages of CBASP therapy. On the basis of the participants’ engagement in this pilot study, it should be examined whether strong therapeutic support at the beginning, as advised in the written information (eg, by planning which modules to be worked on between sessions), can improve IMI uptake and whether intensive blended use toward the end, as observed in this pilot study (especially high in participants 1, 3, 7, and 10), might foster the use of the app as a self-help maintenance treatment after therapy. Monitoring patients’ symptoms could also be a useful feedback system for therapists in making clinical decisions [38] and should be investigated more in the context of blended therapy concepts.

The fact that half of the participants received CBASP group therapy indicates the feasibility of the blended treatment approach in this setting and is consistent with earlier findings of a blended CBT group therapy for depression [61]. Of 6 patients in the blended CBASP group therapy, 2 (33%) showed high use, which, in comparison with the individual setting (5/6, 83% of patients showed high use), could indicate that the blended group therapy may be less able to encourage continuous use. Given the relatively small number of CBASP therapists, blended group therapy could be especially relevant because of higher scalability.

Replacing up to two-thirds of the face-to-face contacts with IMIs use was found to be noninferior to standard CBT treatment [62]. Therefore, blended CBASP therapy might also be promising when it comes to counteracting the treatment gap by allowing therapists to treat a larger number of patients.

As CBASP is also offered in inpatient settings [20,60], blended use as part of inpatient treatment seems promising. The use of *CBASPath* as a self-help tool after successful blended therapy or for bridging therapy breaks, especially after comparatively short inpatient stays, also seems useful and should be investigated in further studies.

During recruitment, we observed that only a few interested therapists were willing to test *CBASPath* with patients. Therefore, a therapist’s assessment of the feasibility of blended therapy and satisfaction with *CBASPath* should be considered to prevent potential difficulties in future studies and subsequent implementation. Further acceptance-facilitating interventions (eg, informational videos) have been proven to be effective in increasing psychotherapists’ acceptance of blended therapy [63] and might help attract therapists.

### Conclusions

The novel treatment approach presented here could allow further optimization of an already effective CBASP treatment and provide patients with a feasible and assessable treatment program. The blended setting itself is particularly coherent with CBASP therapy, despite its highly interactional character. However, the right frequency and optimal embedding should be further investigated to combine the best of the analog and digital worlds**.** Randomized controlled studies are now vigorously needed to investigate the efficacy of blended CBASP therapy and the *CBASPath* tool, with a focus on long-term follow-up to examine long-term responses. If positive, *CBASPath* could help optimize CBASP treatment in the long term and reduce relapses by intensifying therapy and providing patients with PDD with long-term therapeutic support through the app.

## Appendix

Multimedia Appendix 1 Participants’ attitudes toward internet- and mobile-based interventions at baseline.

Multimedia Appendix 2 Participants’ ratings on usability, quality, and satisfaction with CBASPath after 12 weeks of use.

## Abbreviations

APOI: Attitudes Toward Psychological Online Interventions Questionnaire
CBASP: Cognitive Behavioral Analysis System of Psychotherapy
CBT: cognitive behavioral therapy
CSQ-I: Client Satisfaction Questionnaire adapted for internet-based Interventions
IDE: interpersonal discrimination exercise
IMI: internet- and mobile-based intervention
PDD: persistent depressive disorder
RCT: randomized controlled trial
SUS: System Usability Scale
uMARS: user version of Mobile App Rating Scale
