# Supplementary material for: Adding an App-Based Intervention to the Cognitive Behavioral Analysis System of Psychotherapy in Routine Outpatient Psychotherapy Treatment: Proof-of-Concept Study
Source: JMIR Form Res. 2022 Aug 9;6(8):e35482. doi: 10.2196/35482 (PMC9399836; doi:10.2196/35482)
Supplement: Multimedia Appendix 1 [file formative_v6i8e35482_app1.docx]

Appendix 1. Participants’ attitude towards IMIs at baseline

| Participant | APOI total | APOI scepticism and perception of risk | APOI confidence in effectiveness | APOI technologization threat | APOI anonymity benefits |
| --- | --- | --- | --- | --- | --- |
| Participant 1 | 49.00 | 12.00 | 16.00 | 11.00 | 10.00 |
| Participant 2 | 56.00 | 15.00 | 15.00 | 13.00 | 13.00 |
| Participant 3 | 41.00 | 10.00 | 12.00 | 9.00 | 10.00 |
| Participant 4 | 50.00 | 14.00 | 16.00 | 10.00 | 10.00 |
| Participant 5 | 53.00 | 15.00 | 15.00 | 10.00 | 13.00 |
| Participant 6 | 45.00 | 13.00 | 15.00 | 7.00 | 10.00 |
| Participant 7 | 47.00 | 11.00 | 18.00 | 6.00 | 12.00 |
| Participant 8 | 46.00 | 15.00 | 14.00 | 12.00 | 5.00 |
| Participant 9 | 48.00 | 15.00 | 16.00 | 8.00 | 9.00 |
| Participant 10 | 47.00 | 12.00 | 18.00 | 7.00 | 10.00 |
| Participant 11 | 48.00 | 10.00 | 18.00 | 5.00 | 15.00 |
| Participant 12 | 50.00 | 15.00 | 13.00 | 11.00 | 11.00 |
| M (SD) | 48.33 (3.82) | 13.08 (2.02) | 15.50 (1.93) | 9.08 (2.50) | 10.67 (2.50) |

Note*.* APOI = Attitudes towards Psychological Online Interventions; theoretical range of the total score16-80, theoretical range of subscales 4-20
