# Supplementary material for: Adding an App-Based Intervention to the Cognitive Behavioral Analysis System of Psychotherapy in Routine Outpatient Psychotherapy Treatment: Proof-of-Concept Study
Source: JMIR Form Res. 2022 Aug 9;6(8):e35482. doi: 10.2196/35482 (PMC9399836; doi:10.2196/35482)
Supplement: Multimedia Appendix 2 [file formative_v6i8e35482_app2.docx]

Appendix 2. Participants’ rating on usability, quality, and satisfaction with CBASPath after 12 weeks of use

| Participant | SUS total | CSO-I total | UMARS total | UMMARS Engagement | UMARS function | UMARS aesthetics | UMARS information quality | UMARS subjective app-quality |
| --- | --- | --- | --- | --- | --- | --- | --- | --- |
| Participant 1 | 82.50 | 28.00 | 4.10 | 4.40 | 4.00 | 4.00 | 4.00 | 3.50 |
| Participant 2 | 87.50 | 29.00 | 3.83 | 3.00 | 4.00 | 4.33 | 4.00 | 4.00 |
| Participant 3 | 95.00 | 28.00 | 4.08 | 3.80 | 4,00 | 4.00 | 4.50 | 4.00 |
| Participant 4 | 90.00 | 28.00 | 4.17 | 3.60 | 4.75 | 4.33 | 4.00 | 3.75 |
| Participant 5 | 57.50 | 24.00 | 3.78 | 3.60 | 4.00 | 4.00 | 3.50 | 3.50 |
| Participant 6 | 80.00 | 29.00 | 4.11 | 3.60 | 4.50 | 4.33 | 4.00 | 3.75 |
| Participant 7 | 87.50 | 26.00 | 3.88 | 4.00 | 3.75 | 4.00 | 3.75 | 3.00 |
| Participant 8 | 87.50 | 31.00 | 4.34 | 4.60 | 4.25 | 4.00 | 4.50 | 3.75 |
| Participant 9 | 75.00 | 29.00 | 3.60 | 3.40 | 3.00 | 4.00 | 4.00 | 4.00 |
| Participant 10 | 97.50 | 30.00 | 4.56 | 4.40 | 5.00 | 4.33 | 4.50 | 3.50 |
| Participant 11 | 90.00 | 24.00 | 3.51 | 3.20 | 3.50 | 3.33 | 4.00 | 3.75 |
| Participant 12 | 92.50 | 30.00 | 3.98 | 4.00 | 4.50 | 3.67 | 3.75 | 3.50 |
| M (SD) | 85.21 (10.74) | 28.00 (2.26) | 3.99 (0.30) | 3.80  (0.50) | 4.10  (0.55) | 4.03  (0.30) | 4.04  (0.32) | 3.67  (0.29) |

Note. SUS= System Usability Scale, theoretical range of total score 0-100, uMARS= Mobile Application Rating Scale- user version, theoretical range of total scale and subscales 1-5, CSQ-I= Client Satisfaction Questionnaire adapted to Internet-based interventions, theoretical range of total scale 8-32
